# The “Emorality” of Caring: Validation of an Empirical Model of the Moral Feelings of Affective Care in Teaching Communities

**DOI:** 10.3390/bs14110983

**Published:** 2024-10-23

**Authors:** Antonio Rodríguez-Hernández, Joaquín Sepúlveda-Aravena, Mariela Melgarejo-Coronel, Isabel Duarte Lores

**Affiliations:** 1 Department of Developmental and Educational Psychology, Faculty of Psychology and Speech Therapy, University of La Laguna, 38200 San Cristóbal de La Laguna, Spain; antrodri@ull.edu.es; 2 Center for Emotional Education & Psychotherapy—CEDEMOP, Talca 3460000, Chile; mariela.melgarejo@cedemop.com; 3 Department of Clinical Psychology, Psychobiology and Methodology, Faculty of Psychology and Speech Therapy, University of La Laguna, 38200 San Cristóbal de La Laguna, Spain; iduartel@ull.edu.es

**Keywords:** emorality, affective care, moral feelings, resilience, teachers’ emotions, SEMORCUNA

## Abstract

This article presents a study that addresses the challenge of establishing a relationship between the axiological and the affective, by validating a structural model through an assessment instrument (SEMORCUNA) that isolates the moral feelings associated with ‘affective care’. The research sample consisted of 222 teachers, all of whom were either in training or were active professionals in the teaching field. To achieve the research objectives, a group of experts selected a total of 11 moral sentiments, based on which Principal Component Analysis was conducted. Subsequently, Cronbach’s alpha was calculated to determine the internal consistency of the factors obtained. Confirmatory factor analysis was also performed. The results indicate that the selected feelings are part of a single factor. We conclude that all the emotional–moral experiences included in the test are empirically associated with the value of affective care. This work provides a tool to study the degree of teacher identification with the moral feelings that characterize the school as a ‘learning caring institution’, which is a fundamental condition for ensuring ‘resilient educational communities’.

## 1. Introduction

Never have we had so many means and resources to address our existence as a species, but, paradoxically, we have never been so vulnerable to the structural adversities with which we have to coexist. This problematic reality, which is of a systemic nature, therefore, demands a resilient community alternative that goes beyond an individual approach. This work aims to provide an empirical contribution, by applying this social perspective to teacher resilience [1] and teacher wellbeing [2,3].

Rodríguez and Batista [4] have formulated the neologism “emorality” to refer to the theoretical construct with which we can conceptually integrate the set of interrelationships that, as an axiological–emotional network, weave the identity support upon which we build our humanity. The importance of this construct lies not only in its analytical and interpretative utility for better understanding the social and affective dimension of human beings, but it also has practical implications in the field of educational communities. It is fundamental in the development of emotional education, social–emotional learning, and values education, as well as being a tool to strengthen teacher resilience and wellbeing in the face of various adverse contexts. This theoretical construct can be manifested through an alternative value that we have denominated as affective care, which is defined as the disposition to intentional action by individuals belonging to a collective, a community, or society in general, to carry out actions oriented to make its members feel that they are loved and protected. This complex value translates into a series of moral feelings with axiological content, as they transcend individual emotional experiences to connect with more specific values that promote personal and social fulfillment, which are all aligned with the purpose of emotionally protecting and caring for others.

We want to emphasize the community’s sense of this value through its formulation in plural (caring for each other), once we understand that by doing so, we are advocating for the need to address its practical translation in a collaborative manner. We defend the need to overcome the perverse privatization approach to distress that restricts the perspective to self-care [5], undertaking this task within the framework of a group of people united by emotional bonds, who develop, on a daily basis, emotional support. At a contextual level, it has been identified that positive relationships with school administrators, colleagues, family, and friends, are relevant protective factors for teacher resilience [6,7], where we maintain that affective care plays a role in the generation, maintenance, and strengthening of these relationships, potentially influencing teacher wellbeing.

With this contribution, we aim to isolate a series of moral feelings of affective care, from the perspective of teachers, in order to analyze their identification with these feelings and how, in their opinion, they characterize their educational communities. But first, let us address the delineation of the two theoretical foundations of affective care: on one hand, the psychology of care, and, on the other, moral feelings.

### 1.1. The Psychology of Care

The psychology of care has been historically evidenced in the psychology of moral development, family psychology, and organizational psychology. In this sense, we identify the psychology of care as an area of need in educational communities, both to reduce mental health problems (for example, burnout, compassion fatigue, bullying), as well as to promote the wellbeing of teachers, students, and families.

In psychology, the study of care as part of moral development was introduced by psychologist Carol Gilligan, in terms of the concept of the ethics of care [8]. She suggests that care is the foundation of development and supports the connection through emotional bonds of closeness established with others (people and animals). Later, Noddings [9] deepened the explanation, asserting that the ethics of care requires individuals to act with care, which effectively means that we act in a correct or permissible manner when our actions express or demonstrate a motivation of care towards others. In this regard, the ethics of care confronts two types of attitudes: on one hand, an attitude that implies morally and socially valued actions, and, on the other hand, an attitude of indifference or hostility.

In family psychology, care is seen as a behavior that expresses relational values, which emerge from emotions and feelings, making relationships meaningful [10]. In organizational psychology, there is the study of Perceived Organizational Care (POC), defined as an organization-focused concept that reflects the individual employee’s perception of the extent to which their interests, satisfaction, contribution, and professional development and wellbeing, are respected and valued by an organization at large [11].

On the other hand, the need to deepen the study of care in education has been raised [12]. In this context, it has been reviewed from a contemplative approach, a perspective that recognizes the value of cultivating the integrity of the self as a basis for living ethically in the world with others [13]. Wilde [13] argues that concrete caring actions stem from socio-affective awareness, as it facilitates the perception of one’s own affective needs and those of others. In this sense, care involves the emotional experience that drives its action. Similarly, in teachers, care involves attention, trust, the ability to respond to needs, the adoption of narrative nuances, and the cultivation of affectionate relationships with other teachers and with students [14]. In this context, care can take different forms, such as feeling responsible for the success of students [15], teaching to reduce anxiety [16], using motivational strategies [17], and meeting cultural and social needs [18].

Noddings [19] argues for the need for education that is based on the balance between reason and the heart. It is at this point that a bridge is established between the pedagogy of care, emotions, and feelings, in such a way that the idea of affective care becomes an essential axis of the teaching–learning activity that involves the teacher’s person [4,20].

Therefore, if the action of caring is based on the emotional bonds established between individuals, it seems necessary, for an understanding of the psychological processes associated with affective care and, thus, as a contributor to the psychology of care [21], to create an empirical construct that isolates the affective dimension on a more transcendent plane than the emotional experience itself. For this purpose, in the present research, a selection of different moral feelings has been chosen.

### 1.2. Moral Feelings

For centuries, several authors have contributed to the discussion on the relationship between emotion and morality. In moral philosophy, Aristotle refers to eudaimonia as the result of a morally virtuous life. Similarly, in 1677, Baruch Spinoza stated that feelings are the basis of moral behavior; a theory that influenced Damasio [22] to start analyzing and highlighting the convergence between morality and emotion from the perspective of neuroscience. In 1759, Adam Smith [23] explored the relationship between feelings and morality in his book “*The Theory of Moral Sentiments*”. Kant, in 1785, in one of his works, identified “do no harm” as a fundamental moral principle [24]. During the 20th century, from the perspective of moral development psychology with a rationalist and cognitive–evolutionary approach, Piaget [25] and Kohlberg [26,27] theorized about the development of moral criteria and moral reasoning at different developmental stages. In turn, from the perspective of sociology, Durkheim addressed the importance of moral education in social cohesion and in the formation of a collective conscience, in his work “*Moral Education*” [28]. He argued that moral education is fundamental to maintaining stability and order in society, as it promotes shared values and social norms that unite individuals into a community. Durkheim highlighted the importance of education in transmitting moral norms and in shaping responsible and ethical citizens.

Moral feelings, which in the scientific literature are often interchangeably called moral emotions, have been a topic of interest in scientific research due to their influence on social and moral development and because they are involved in most ethical decisions and actions [29,30]. The objective of moral feelings is not only to preserve social relationships [31], but also to facilitate prosocial behaviors [32].

One of the approaches that has emerged came from psychologist, neuroscientist, and philosopher Joshua Greene, who proposed the dual-process theory [33,34,35,36], asserting the existence of two different systems in the brain: one of these systems is responsible for emotionally charged responses and the other system is responsible for responses based on consequences, grounded in reason.

On the other hand, social intuitionism, supported by Jonathan Haidt [37,38], suggests that moral judgments stem from intuition, with a high emotional component, and, once made, people use reasoning to justify them. Therefore, moral judgments are not the result of reason, but of emotions and feelings [39]. In this way, moral emotions can be useful in motivating morally significant actions [40,41,42,43].

Likewise, Jesse Prinz [44], who has shown interest in the link between emotion, moral psychology, and consciousness, proposes an emotionalist–sentimentalist perspective and establishes the Constitution Model, according to which morality primarily involves emotions and feelings, and that moral judgments have an emotional character [45]. However, his approach seems to present an inconsistency in the way it exposes the relationship between moral norms and feelings. Nevertheless, Prinz [46] differentiates between moral feelings depending on the transgressor and the type of transgression. On one hand, he presents reactive feelings that are directed towards other people, which can be classified into: (a) approval, praise, commendation, etc.; and (b) disapproval, guilt, indignation, contempt, aversion, etc. On the other hand, there are reflective feelings, which are directed towards oneself for the moral harm that has been committed. However, when there are no transgressions and a person’s actions align with their moral principles, pleasant/prosocial moral feelings arise, depending on who performs and who receives the moral action, namely admiration, gratitude, gratification, and dignity.

As a proposal for moral feelings provoked by the violation of certain ethical codes, the CAD model proposed by Rozin et al. suggests a triad of contempt, anger, and disgust based on the type of moral violation committed: if social or community norms are breached, contempt arises; if the infraction involves physical harm, anger is generated; if there is a transgression against norms related to cleanliness and hygiene, the feeling is disgust [47]. Thus, it can be understood that moral feelings are those experienced by an individual when facing the prospect of a moral principle, that is, when based on moral categories developed by society [48]. Therefore, for a feeling to be moral, moral and normative standards must be taken into account, which means considering beliefs about non-compliance with standards and, at the same time, referring to the stereotypes inherent to individual codes and beliefs [49]. On the other hand, it is believed that moral feelings are the motivational forces that make it possible to do “good” and avoid “evil” [50,51,52]. These forces depend not only on intrapersonal factors, but also on the trajectories of development throughout life, as proposed by the Model of the Development of Moral Emotions [53,54]. This perspective highlights the complex interplay between individual emotional development and moral behavior, suggesting that our capacity for moral feelings evolves over time, influenced by both personal experiences and broader social and cultural contexts.

Conill and García-Marzá [55] argue that the affective dimension is key to understanding and applying moral knowledge and its potential for action, as well as its transformation of resources into a possibility for action. That is, emotions and moral feelings are the foundation of that moral behavior that leads us to behave in a certain way. In this sense, it could be said that moral feelings serve the function of uniting individuals through a bond of interdependence, allowing for the recognition of others and, reciprocally, attributing to them the same human dignity and vulnerability [56]. This perspective emphasizes the importance of emotions in moral decision-making and ethical behavior, highlighting how our feelings toward others and situations influence our actions and contribute to the formation of moral communities. Indeed, moral feelings, such as compassion or guilt, are considered emotions that influence a person’s understanding of the normative nature of standards related to the wellbeing and care of another person, in terms of an eudaimonistic judgment [57,58]. They reflect internalized principles regarding the welfare of others, justice, and equity, acting as virtuous feelings [59]. This perspective underscores the role of moral emotions in guiding ethical behavior and decision-making, highlighting their importance in fostering a sense of responsibility and care for the wellbeing of others within a moral and ethical framework.

Like other emotions and feelings, moral feelings arise in response to the evaluation of a stimulus or event [60,61], evoking physiological responses, such as changes in a person’s heart rate [62] and skin conductance [63]. Unlike basic emotions, moral feelings often require self-reflection [64], social cognitive skills, such as the theory of the mind [65], and the ability to balance goals, perspectives, and feelings oriented towards oneself and others [66]. This highlights the complex nature of moral feelings, encompassing both physiological and cognitive dimensions. This means that moral feelings are similar to basic emotions and other non-morally significant emotions in terms of their ontogeny, but they differ in terms of their cognitive and contextual triggers. For example, compassion may feel similar to experiencing sadness. However, compassion is associated with morally relevant contexts involving another person who is facing a problem, whereas sadness is less tied to a specific context. In the case of guilt, it has been observed that it can facilitate subsequent prosocial behavior, starting with an apology [67].

In empirical research conducted with teachers, it was found that when resolving a moral conflict involving their students, teachers who were experiencing prosocial feelings tended to resolve the dilemma with an attitude of solidarity and care, driven by a generosity to help their students, which in turn impacted their affective wellbeing [68]. This study highlights the impact of prosocial emotions on decision-making in regard to moral dilemmas, suggesting that such feelings can lead to more compassionate and caring outcomes, benefiting both the helper and the recipient. On the other hand, teachers experiencing unpleasant affectivity tend to make decisions based on self-protection and withdrawal, delegating the issue to others. In this sense, moral feelings, such as empathy, compassion, forgiveness, and gratitude, among others, can drive behaviors that create bonds of care between teachers and their students, which are related to their strengths and competencies. This underscores the importance of fostering positive emotional environments in educational settings, not only for the wellbeing of students, but also for enhancing the moral and professional capacities of teachers.

After an extensive review of the scientific literature, we found that various moral feelings have been separated into different categories:

- Emotions of appreciation [69];
- True feelings [70,71];
- Positive/pleasant/pleasurable emotions [72,73,74,75];
- Self-conscious emotions [76];
- Transcendent emotions [77,78,79];
- Laudatory emotions or emotions related to praise [80];
- Positive moral emotions [81,82];
- Corrosive emotions [83];
- Prosocial emotions [84];
- Transformative feelings [4].

These categories illustrate the wide range of emotions and feelings that play a role in our moral lives and interactions with others. They highlight the complexity of the human emotional experience and its crucial role in shaping ethical behavior and social relationships.

It is noted that the majority of studies focusing on the category and quality of moral emotions or feelings are centered on students (children and adolescents). For instance, research has been carried out to investigate emotions such as shame [85], as well as pride, guilt, and shame, in early childhood, among children with autism spectrum disorder [86]. Additionally, studies have looked into pride, guilt, and shame in early childhood, as reported by their parental figures or caregivers [87,88]; guilt and adaptive anger [89,90]; sympathy and prosociality in children [91]; guilt and cooperation [92]; empathy in early childhood in children with autism spectrum disorder [93]; respect in children and adolescents [94]; empathy in primary and secondary school students [95,96]; authentic moral pride in adolescents [97]; and benign and malignant envy in adolescents [98]. While, in adults, benign and malignant envy have been studied [99]; as well as pride, love, compassion, and wonder [100,101].

### 1.3. The Assessment of Moral Feelings

From a review of the instruments used to empirically isolate moral sentiments, we contribute those that have been evaluated through the questionnaire validated in this work.

Authenticity is defined as the degree to which one behaves in accordance with what they consider to be their true or genuine self: who one “is” as a person [102]. In affective terms, this would be a true emotional experience regarding an event [103] or the feeling that an individual is being their true self [104,105]. It has been evaluated as a character strength, a positive personality trait, in terms of the VIA-S, which measures a total of 24 strengths [106]. It has also been assessed as a personality trait in terms of the Authenticity Scale, which is a 12-item measure designed to assess a tripartite conception of authenticity [107,108]. The measure consists of three sub-scales, with four items each: authentic living (e.g., “I always stand by what I believe in”), self-alienation (e.g., “I feel alienated from myself”), and acceptance of external influence (e.g., “Other people influence me a lot”). Another instrument is the Southampton Authenticity Scale—SAS [109]. There is no measure for a moral feeling.

For kindness, there is a specific measure created by Youngs, Yaneva, and Canter [110]. Two facets of kindness are measured: one, the psychological source of action (based on principles or empathy) and, the other, the form of expression (through psychological involvement or following a social prescription). Different modes of kindness that are part of these two facets are evaluated: principle-prescribed social kindness; principle proactive kindness; affective proactive kindness; and affective socially prescribed kindness. These modes respond to a central theme of kindness: anthropophilia. The instrument has a total of 45 items.

For compassion in preschool teachers, the Adult Compassion Scale has been used, which consists of 20 items grouped into four dimensions: behavioral compassion, emotional compassion, cognitive compassion, and motivational compassion [111,112]. Additionally, the Santa Clara Brief Compassion Scale was used in Finland [113].

Regarding trustworthiness, studies have been conducted to analyze it in close relationships [114]; in regard to the interaction with institutions [115]; in relation to other moral feelings [116]; and in the relationship between adults and adolescents [117]. This feeling is associated with a set of behaviors of dependence on another, a belief about the future behavior of another person, an abstract mental attitude that another is trustworthy, a feeling of confidence accompanied by security, and a complex neuronal process that includes various feelings [118]. The operationalization of trustworthiness, as described by Tschannen-Moran and Hoy, involves five facets: (a) benevolence, understanding the interests of others and prioritizing their needs over one’s own; (b) reliability, demonstrating positive and predictable behavior; (c) honesty, keeping one’s word, reporting facts truthfully, and taking responsibility for mistakes; (d) openness, exchanging information based on goodwill; and (e) competence, possessing the necessary skills to meet expectations [119]. This has been qualitatively studied using semi-structured interviews and focus groups [120].

Empathy in teachers, as described by Coplan [121] and Hong et al. [122], is the ability to understand and share the feelings and perspectives of their students. Teacher empathy consists of both interpersonal and social empathy, blending the role of the teacher [123,124]. This includes the teacher’s ability to recognize and address the emotional needs of their students and to adjust their teaching and guidance accordingly [125]. The impact of empathy on teacher–student relationships is crucial for achieving positive student outcomes [126,127,128]. To measure this emotional competency, the Interpersonal Reactivity Index (IRI) has been used, conceptualizing empathy as a multidimensional construct, namely made up of affective empathy and cognitive empathy, and assesses dispositional empathy [129,130]. Another instrument employed is the Questionnaire of Cognitive and Affective Empathy—QCAE [131]. For teachers in training, the Cognitive and Affective Empathy Test (TECA) has been used, for example, in Colombia and Mexico [132,133,134].

To measure generosity, Smith and Hill developed the Interpersonal Generosity Scale (IGS), which includes four characteristics [135]. Firstly, it involves social relationships. Secondly, it focuses on giving behavior. Thirdly, it concerns personal, irreplaceable goods. Fourthly, it is not necessary to be purely altruistic. Six dimensions of generosity are evaluated, namely (1) attention, (2) compassion, (3) generosity, (4) personal extension, (5) courage, and (6) verbal expression. The preliminary version of the tool contained 26 items. After reviewing its validity and reliability as applied to over 2000 participants in 2 surveys, the final 10 items emerged. Dwidienawati et al. confirmed its reliability and validity in the Indonesian population, highlighting the ease of understanding the items, even when translated [136].

To assess gratitude, the revised Gratitude, Resentment, and Appreciation Test (GRAT-R) has been used [137,138,139]. It is a tool used to measure the disposition to gratitude and consists of 44 questions that include three categories of gratitude: a sense of abundance (i.e., “life has been good to me”), the appreciation of simple pleasures (i.e., “I really enjoy the change of seasons”), and social appreciation (i.e., “I am very grateful for my friends and family”). Another instrument used to assess dispositional gratitude is the Gratitude Questionnaire (GQ-6), which is composed of six items, and has been validated in adult and adolescent populations [140,141]. Although the GQ-6 is the most commonly used instrument, there are other tools available for assessing gratitude. These include the Gratitude Adjective Checklist (GAC), used to measure gratitude as an emotion, mood, or disposition [142,143]; the Gratitude Questionnaire-20 items (G20) [144]; the Gratitude Scale [145]; and the Work Gratitude Scale, used to measure gratitude in the workplace [146].

Forgiveness, understood as a multidimensional process that includes cognitive, emotional, motivational, and social characteristics [147,148,149,150], has been measured with the Heartland Forgiveness Scale (HSF) [151,152,153,154]. The scale contains six items that assess self-forgiveness, six items that assess forgiveness of others, and six items that assess forgiveness in situations. For teachers, the self-forgiveness dimension shows that teachers can console themselves without any resentment for the negativity and mistakes they have experienced. The forgiveness of others dimension shows that teachers do not act strictly against students regarding the mistakes they make; instead, they seek to understand them, they do not wish to punish them, and can move past their frustrations towards their students. In regard to the forgiveness in situations dimension, it refers to teachers not getting trapped in negative thoughts regarding uncontrollable negative situations they experience in school and, ultimately, moving away from negative feelings. It has been found that forgiveness in teachers is a predictor of teaching self-efficacy [155,156].

Respect has been studied in children and adolescents using semi-structured interviews and incidental observations [157,158,159]. In another study, the Perceived Respect Scale adapted to the school context was used, consisting of six items that measure to what extent students feel that teachers behave respectfully towards them [160].

Solidarity has been evaluated as emotional solidarity through the use of focus groups [161]. There is a lack of empirical measurements in an educational context from the perspective of affectivity.

Finally, emotional engagement has been studied in students as a sense of belonging to a peer group [162,163] and, in terms of teachers, as a socio-emotional bonding competence [164,165].

Morality has its origin in experiences involving the emotions and feelings of people. A review conducted by Ellemers et al. analyzes empirical studies on morality, pointing out a gap between theoretical perspectives and empirical research, especially regarding moral emotions; it suggests that moral emotions are underexplored in research, despite their fundamental role in moral behavior and decision-making [166]. This gap indicates the need to continue studying moral feelings, including those experienced by teachers. Indeed, the teaching profession is considered a moral profession and it cannot be sustained unless it combines rational principles with ethical behavior, which in turn involves feelings and emotions. Scientific research on the moral feelings of teachers has been found to be scarce. Furthermore, there are no studies or instruments that specifically group and identify the moral feelings investigated in our study. Although the study of moral feelings is based on the preservation of social bonds or relationships, to date, there has been no in-depth exploration of specific clusters of affective care and moral feelings.

In our research, a selection of moral feelings associated with affective care has been made. The following Moral Feelings of Affective Care—SEMORCUNA (the acronym is derived from the Spanish name for Sentimientos Morales del Cuidarnos Afectivo) will be studied: authenticity [167,168,169,170]; kindness [171,172,173]; compassion [174,175]; trustworthiness [114,115,116,117,118,119,120]; empathy [121,122,123,124,125,126,127,128,129,130,131,132,133,134]; generosity [176,177]; gratitude [178]; forgiveness [147,148,149,150,151,152,153,154,155,156]; respect [157,158,159,160]; solidarity [179,180]; and emotional engagement [164,165]

The objective of this work is to contrast the existence of the value of “affective care” by validating and applying an instrument (SEMORCUNA) that isolates the moral feelings associated with this value, so that we can study the degree of identification with them by teachers and how they perceive these feelings in the educational context, in order to characterize them as “communities of care”.

## 2. Materials and Methods

### 2.1. Participants

The total sample obtained in the research consisted of 253 individuals, of which 67.6% were women, 32% were men, and 0.4% selected the “other” option, with an average age of 30.76 years (SD = 13.147; range = 17–62). A total of 96.4% of the sample were from the Canary Islands Autonomous Community (85.8% from Santa Cruz de Tenerife and 10.7% from Las Palmas), while 3.6% resided in other Spanish Autonomous Communities (e.g., Valencia, Galicia, Aragon). All participants were linked to the teaching profession, either at the training stage (61.7%) or as active workers (38.3%). The majority of individuals were pursuing or had a degree in primary education teaching (43.3%), while the rest had or were pursuing other university studies (e.g., biology degree, mathematics degree, a Bachelor’s or a degree in English studies). Among the trainee teachers (*n* = 156), 31.2% were in training as secondary education teachers and 30.4% as primary education teachers.

Among the active teaching professionals (*n* = 97), the majority were teachers (84.5%) in the primary education stage (43.4%) in a public school (70.1%), as shown in Table 1. The average teaching experience was 15.61 years (SD = 10.450) and the majority had received training in emotional education (61.9%), through courses of more than 30 h (21.6%).

### 2.2. Instrument

The measure used is the Moral Feelings of Affective Care (SEMORCUNA) questionnaire (the acronym is derived from the Spanish name for Sentimientos Morales del Cuidarnos Afectivo). As part of its construction, the questionnaire was administered to a group of 17 experts (14 women and 3 men) with an average age of 49, of Spanish (12), Mexican (1), Argentinean (2), Paraguayan (1), and Colombian (1) nationality. All of them were either graduates in higher education studies (psychology, pedagogy, psychopedagogy, philology, or law) or had Master’s and/or doctoral degrees. The experts were selected based on convenience sampling, as they were part of the research group to which the authors belonged, or were academics and/or professionals with a track record of studying the subject under investigation.

Through a Google form, they were presented with 15 moral feelings, along with their names and definitions extracted from the scientific literature (this information is presented in Appendix A).

After defining what was meant by affective care (the intentional disposition of individuals belonging to a group, a community, or society in general, to carry out actions aimed at making their members feel loved and protected), they were asked to rate on a scale ranging from 1 to 7 (where 1 means little and 7 represents much) how much they considered each feeling to be related to affective care, as previously defined. Additionally, they were asked to provide any necessary observations for each moral feeling to improve the wording.

The objective of this procedure was not to establish an agreement among the judges, but to select, from the total moral feelings presented for evaluation by the experts, those that they believed were most related to affective care based on the weight assigned to them. Therefore, a restrictive average score was chosen to determine the selection, which in this case was 6 or higher (remember that the scale range was 1–7). In addition to this quantitative evaluation, it was of interest to gather qualitative observations regarding the wording on the moral feelings in order to enhance them. In this way, after completing this procedure, 11 moral feelings were selected; their definitions and average rating scores from the judges are presented in Appendix B.

Following on from this, the instrument to be validated was constructed, consisting of the following sections. Firstly, a series of items that collected the participants’ referential data were established. Subsequently, they were presented with instructions on how to complete the questionnaire. Next, the 11 moral feelings selected by the judges, along with their respective definitions, were presented for the participants to assess the extent to which they identified with each of these feelings using a scale from 1 to 7 (where 1 is little and 7 represents much).

It should be noted that the instrument included another series of items that are not the focus of study in this work.

### 2.3. Procedures

The questionnaires were distributed via a Google form to active teachers who were available and chose to participate voluntarily. The questionnaires were completed voluntarily and ethical guarantees, such as consent, anonymity of the participants, and data privacy, were ensured. The data were kept confidential, under the institutional custody of the research group.

Additionally, the questionnaires were distributed in the same manner to students enrolled in the primary education teacher program and the Master’s in secondary education teacher training program. The data obtained were transferred to an Excel template for subsequent inclusion in the SPSS v.26 statistical program database, wherein the Exploratory Factor Analysis (EFA) and Cronbach’s alpha coefficient analysis were conducted. The structural equation modeling software AMOS v.23 was used for the confirmatory analysis.

### 2.4. Statistical Analysis

To address the research objectives, Principal Component Analysis was conducted to extract the structure of the factors from the instrument. Subsequently, Cronbach’s alpha was calculated to determine the internal consistency of the factors obtained. Confirmatory factor analysis was also performed, including the scoring of the questionnaire variables using the participant sample in the study. All these analyses were carried out using the statistical package SPSS v.26 and the R software v. 4.4.1 and the ULLRToolbox for the confirmatory factor analysis.

## 3. Results

### 3.1. Data Cleaning

Before starting the data analysis to test the questionnaire’s factorial structure, the database was cleaned to prevent potential distortions in the results. All the procedures described were conducted using the statistical software SPSS v.26.

Initially, 22 participants were excluded for not fully completing the SEMORCUNA questionnaire, resulting in a sample of 231 individuals. Additionally, outliers were removed using the Mahalanobis distance method and the Chi-squared test. The sample without missing values initially consisted of 231 participants, which was reduced to 222 after identifying 9 individuals with atypical or extreme scores.

Subsequently, the nature of the data was analyzed to determine the type of analysis to apply and whether variable transformation was necessary. The examination of item normality was based on skewness and kurtosis z-scores, which confirmed the data normality as all the statistics fell within the range of ±1.96, as shown in Table 2.

The only statistics outside the specified range were the kurtosis of the “kindness” and “trustworthiness” items. However, since the skewness met the criterion, the data normality was considered valid.

### 3.2. Exploratory Factor Analysis

The first step to understand the internal structure of the SEMORCUNA questionnaire was to conduct Exploratory Factor Analysis (EFA). The varimax rotation was used to predict the factor independence, along with the unweighted least squares extraction method, which is recommended for Likert-type scales. Initially, EFA was performed with the complete sample, followed by separate EFAs for active teachers and teachers in training.

For the complete sample (*N* = 222), an adequate KMO index (0.885) was observed, and Bartlett’s test was significant (X2(55) = 893.580, *p* < 0.001), indicating that the variables were related to each other, making it appropriate to conduct the EFA to analyze the underlying structure of the data [181].

Regarding the number of factors extracted from the analysis, using the total sample of teachers, a single factor was observed, indicating the unidimensionality of the construct, with an eigenvalue of 4.982 and a variance percentage of 45. Table 3 shows the factor loadings of the different items, which are above 0.30, so are considered adequate [182].

Next, the process was repeated with the sample divided based on the participants’ career stage, maintaining the same conditions described earlier for the EFA. In this instance, both EFAs showed the presence of two distinct factors, with the factor using the sample of teachers in training being more consistent.

The EFA using the sample of active teachers had a good KMO index (0.804) and a significant Bartlett’s test (X2(55) = 332.443, *p* < 0.001), as did the sample of teachers in training, with an adequate KMO index (0.874) and a significant Bartlett’s test as well (X2(55) = 526.496, *p* < 0.001).

Both factorial solutions yielded two factors, following the application of Kaiser’s rule and a scree plot based on the obtained eigenvalues In the sample of active teachers, the first factor had an eigenvalue of 4.497 (40% variance) and the second factor had an eigenvalue of 1.150 (10% variance). In the sample of teachers in training, the first factor had an eigenvalue of 4.926 (44%) and the second factor had an eigenvalue of 1.112 (10%).

However, when considering the factor loadings found in both analyses, differences in the factor behavior were observed (see Table 4). In the sample of active teachers, the factor loadings for the second factor were all below 0.30, which were not considered appropriate for the factor, while in regard to the first factor, all the items were loaded adequately. In contrast, in the sample of teachers in training, three items were loaded above 0.30 in regard to the second factor. Specifically, the items of authenticity, trustworthiness, and respect had higher loadings, as the rest of the items had higher factor loadings in regard to the first factor. This aligns with the obtained communalities, as these three items have the lowest communalities, along with the item emotional forgiveness, indicating that the variance of these three feelings seems to be explained to a lesser extent by the factors.

To conclude, the reliability of the SEMORCUNA questionnaire was analyzed from two different perspectives: as a unidimensional scale and as a two-factor scale. The decision was made to test the instrument’s reliability from a unidimensional perspective due to the high percentage of variance explained by the first factor and the limited number of items in the second factor, as well as for theoretical reasons.

The reliability of the complete scale showed good internal consistency indices in regard to each of the samples, with a Cronbach’s alpha of 0.87 for the complete sample, 0.84 for active teachers, and 0.86 for teachers in training. Regarding the consistency of the first factor, composed of eight items, good consistency was also observed (complete sample: α = 0.85; active teachers: α = 0.81; teachers in training: α = 0.86). However, the second factor, composed of the items authenticity, trustworthiness, and respect, did not reach the cutoff point of 0.70, established in the literature [183]. It is worth noting that Cronbach’s alpha for the complete sample (α = 0.60) and teachers in training (α = 0.63) was higher than that for active teachers (α = 0.44), which is consistent with the results observed in the EFA.

### 3.3. Confirmatory Factor Analysis

The factorial structure found in the EFA was tested through confirmatory factor analysis (CFA) to validate the instrument. The structural equation modeling software AMOS v.23 was used to test the two factorial structures: a unidimensional one and a two-factor structure grouped under a second-order factor. These structures were tested for the samples of active teachers and teachers in training separately, considering the results observed in the EFA, as well as for the complete sample.

As a prerequisite for estimating the model, the multivariate normality of the data was explored for the complete sample. The Mardia coefficient was 38.316, allowing for analysis as normality was confirmed, since the statistic was below p(p+2) [184]. The maximum likelihood method was used for model estimation due to its robustness, as the Mardia coefficient did not exceed 70 [185].

**Figure 1:**
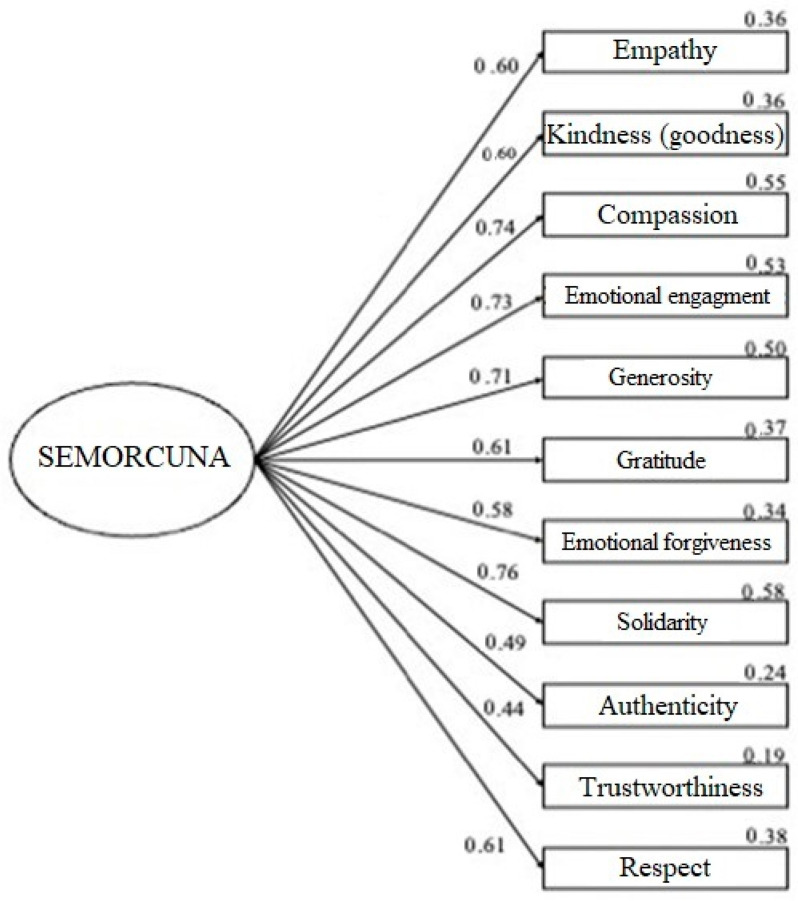
CFA of the unifactorial model for the complete sample. Note: Graphical representation of the model, with standardized factor loading estimates. The errors associated with the items have not been included in the representation for clarity of the model. All lines indicate significant relationships at a level of *p* < 0.001.

Initially, the model of a single factor was tested (Figure 1), supported by the internal consistency of the unidimensional scale (α = 0.87) and the EFA result indicating the presence and greater consistency of a single factor. This model shows good fit indices, according to Hu and Bentler’s criteria, for all the samples (Table 5) [186].

**Figure 2:**
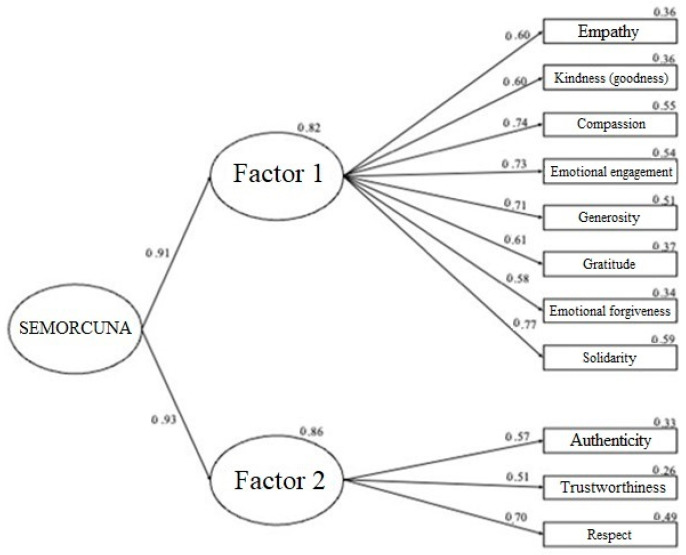
CFA of the bifactorial model for the complete sample. Note: Graphical representation of the model, with standardized factor loading estimates. The errors associated with the items have not been included in the representation for clarity of the model. All lines indicate significant relationships at a level of *p* < 0.001.

The second model tested consisted of a two-factor structure, as indicated by the EFA, grouped under a second-order factor (see Figure 2), to maintain theoretical coherence and a global measure of the construct. This model showed better fit indices than the previous model, suggesting that this structure would be the most suitable for working with the instrument. The only sample where the fit indices worsened with the bifactorial model involved the sample of active teachers, but adequate indices were still obtained for working with this model.

In addition to better goodness-of-fit indices, it is also evident that all the factor loadings are above 0.30, without the need to eliminate any items, and there is an increase in the saturation of the items in terms of the second factor. This version of the model can be considered the best based on the AIC index, which is lower in terms of the two-factor model, indicating a better fit [187], except for the sample of active teachers.

**Table 5:** Goodness-of-fit indices of the models.

	Complete Sample (*N* = 222)		Active Teachers (*n* = 92)		Teachers in Training (*n* = 130)		Acceptance Range
	Unifactorial	Bifactorial	Unifactorial *	Bifactorial	Unifactorial	Bifactorial	
c^2^/*df*	2.422	2.228	1.782	1.667	1.761	1.354	Ideal below 3
CFI	0.927	0.938	0.926	0.903	0.932	0.969	0.90 or higher
GFI	0.929	0.936	0.899	0.887	0.908	0.934	0.90 or higher
RMSEA	0.080	0.075	0.074	0.086	0.077	0.052	Acceptable below 0.10
SRMR	0.045	0.040	0.063	0.064	0.056	0.047	Below 0.08
AIC	150.590	141.824	110.688	117.696	121.474	104.205	Lower index indicates better fit

Note: * These indices are obtained by correlating the errors for the generosity and solidarity items, based on modification indices [188].

## 4. Discussion

In the present research, we have validated an instrument to identify a set of what we have called moral feelings of affective care. The results obtained through confirmatory factor analysis indicate that the selected feelings are part of a single factor. Therefore, as a primary conclusion, we can affirm that all the affective experiences of a moral nature captured by the test are empirically linked to the overall value we sought. This is consistent with the literature related to prosocial moral feelings, suggesting an altruistic prosocial tendency that goes beyond personal benefit for the individual experiencing them.

In the theorization of care, only a few feelings have been highlighted in general, such as compassion [8], empathy [14], care empathy [189,190], and love [191]. Pulcini suggested the need to delve into the emotions and feelings that drive care [192]. Therefore, a contribution by the results of our study is to enrich, demonstrate, and specify the moral feelings of “good” emotional care. The new SEMORCUNA instrument, as far as it has been determined, is the first measurement instrument for moral feelings of affective care.

Since the evaluated sample consisted of individuals from two teaching groups (active and in training), we decided to study whether there were differential factorial models in regard to the two groups. While the unifactorial model was maintained in the sample of active teachers in terms of the total sample, for the teachers in training, both in the exploratory and confirmatory analyses, a second factor consisting of three feelings emerged, namely authenticity, trustworthiness, and respect.

It is important to note that the internal consistency of this second factor, associated with the sample of teachers in training, is insufficient; however, the fit indicators of the second-order model obtained in the confirmatory analysis are appropriate. Consequently, although from a statistical point of view the importance of this structural model can be questioned, we consider that the second identified factor has a theoretical value that should be considered in this discussion. We can hypothesize that the three feelings of the second factor (authenticity, trustworthiness, and respect) share a common thread that distinguishes them from other moral feelings of affective care. What distinguishes these feelings is their inherent connection to establishing a genuine and trustworthy relationship. Authenticity, as a feeling, encompasses the genuine expression of one’s true self, fostering a sense of trust and openness. Trustworthiness, on the other hand, is based on the stable display of honesty and integrity, laying the foundation for a trustworthy and credible relationship. Finally, respect serves as a cornerstone for recognizing the value and dignity of everyone, promoting a culture of acceptance, emotional validation, inclusion, and understanding. Our interpretation is that these are feelings have a quality of prerequisites for affective care, giving them an ethical character in terms of the affective experiences. While the rest of the moral feelings (kindness/goodness, compassion, emotional engagement, empathy, generosity, gratitude, forgiveness, and solidarity) are put into action when manifesting affective care towards others, the coherence experienced in showing and communicating to others how one truly feels (authenticity), the assurance conveyed to another person to establish a sincere relationship with them (trustworthiness), and the consideration for others, leading to valuing and validating their opinions, actions, and feelings (respect), constitute a “pre-care” factor that provides an ethical condition to the other mentioned moral feelings. While ethical or pre-care feelings might be more linked to our broader and permanent reasoning, moral feelings of care would be linked to more specific moral situations.

In other words, a sociopath could mimic being empathetic, generous, or supportive, to achieve immoral ends in their relationships with others. To discern the ethical nature of their affective care intentions, it would be necessary to observe whether these intentions are authentic, reliable, and expressed with respect towards others. Similarly, would we perceive compassion, gratitude, or emotional engagement in the same way if the person exhibiting these feelings was not emotionally authentic, trustworthy, or respectful in their caregiving actions? Although we have not found previous studies supporting these results and despite the lack of sufficient internal consistency at a statistical level for this sub-factor to provide methodological guarantees of its empirical relevance, we believe that its appearance in the sample of teachers in training, with appropriate fit indicators in one of the confirmatory models, provides an interpretative significance that deserves consideration. This is one of the lines of continuity in this work, emphasizing the need to expand the sample of evaluated subjects, both quantitatively and by including other individuals who work in the field of affective care, as well as individuals from the general population who do not engage in such activities professionally. This expansion would allow us to determine whether, in addition to obtaining a general factor of moral feelings, a specific one with this ethical quality can also be isolated.

This study has some limitations. First, the assessment of teachers’ moral feelings is based solely on teachers’ self-reports. Future research could incorporate hetero-assessment to allow comparisons with teachers’ self-reports. Second, due to its cross-sectional design, future research could employ a longitudinal design or intervention studies to explore the maintenance and causal relationships between different feelings of affective care. Third, the sample used in this study may lack representativeness, making it necessary to replicate studies with larger samples and from different countries and cultures. Finally, the study was conducted with a sample of teachers only; however, the function of affective care is exercised by other professionals as well and can be generalized to any group of people who establish emotional bonds to help others. This initial exploration of the value of affective care in primary and secondary school teachers provides an avenue for future studies to continue exploring empirical results on the social meaning of this value in different professional contexts (health workers, companies, university professors, among others). Especially as we continue to face challenging times, inherent to diverse societies and cultures, salutogenic and resilient approaches will be needed to protect ourselves collectively, with “heart” and ethics at the forefront.

## Figures and Tables

**Table 1:** Frequency and percentage of active practicing teachers.

Variables	*n*	%
Professional profile	97	100
Teacher	82	84.5
School counselor	2	2.1
Management team member	9	9.3
Other	4	4.1
The educational stage in which teaching is carried out	97	100
No educational stage	2	2.1
Preschool	5	5.2
Preschool and primary	12	12.4
Primary	42	43.4
Secondary education (ESO)	24	24.7
Secondary education (ESO) and baccalaureate	10	10.3
Baccalaureate	2	2.1
Relationship with the class group	97	100
None	2	2.1
Tutor	31	32.0
Specialist	24	24.7
Tutor and specialist	29	29.9
Other	11	11.3
Type of educational center	97	100
Public	68	70.1
Concerted	27	27.8
Private	2	2.1
Have you received training in emotional education?	97	100
Yes	60	61.9
No	37	38.1
Type of emotional education training received	97	100
Master’s degree/university expert	7	7.2
Course of more than 30 h	21	21.6
Course of less than 30 h	19	19.6
Seminar	12	12.4
None	38	39.2

Note: All individuals agreed to participate voluntarily in the research after providing their informed consent, following being given an outline of the purpose of the study and the features of the measures that they would need to respond to.

**Table 2:** Descriptive statistics of the items in the SEMORCUNA questionnaire.

Items	Factor Loadings
Authenticity	0.503
Kindness (and goodness)	0.605
Compassion	0.733
Emotional engagement	0.727
Trustworthiness	0.450
Empathy	0.596
Generosity	0.700
Gratitude	0.609
Emotional forgiveness	0.587
Respect	0.629
Solidarity	0.753

Note: *N* = 222.

**Table 3:** Factor loadings EFA using the complete sample.

	M	SD	Asymmetry		Kurtosis		Min.	Max.
			Statistic	SD	Statistic	SD		
Authenticity	5.98	0.902	−0.636	0.163	−0.149	0.325	3	7
Kindness (and goodness)	615	0.877	−1.350	0.163	2.853	0.325	2	7
Compassion	6.00	1.000	−0.922	0.163	0.536	0.325	3	7
Emotional engagement	5.93	1.065	−1.124	0.163	1.341	0.325	2	7
Trustworthiness	6.11	0.964	−1.442	0.163	3.180	0.325	2	7
Empathy	6.32	0.873	−1.290	0.163	1.397	0.325	3	7
Generosity	5.97	0.963	−0.643	0.163	−0.269	0.325	3	7
Gratitude	6.10	0.951	−0.901	0.163	0.197	0.325	3	7
Emotional forgiveness	5.33	1.303	−0.601	0.163	−0.224	0.325	2	7
Respect	6.55	0.655	−1.352	0.163	1.417	0.325	4	7
Solidarity	6.10	0.884	−0.839	0.163	0.437	0.325	3	7

Note: *N* = 222.

**Table 4:** Factor loadings and communalities in the EFA.

	Active Teachers (*n* = 92)			Teachers in Training (*n* = 130)		
	Commonality	Factor Loading		Commonality	Factor Loading	
		1	2		1	2
Authenticity	0.323	0.502	−0.150	0.267	0.456	0.308
Kindness (and goodness)	0.412	0.518	0.270	0.432	0.631	0.002
Compassion	0.535	0.723	0.243	0.582	0.768	−0.097
Emotional engagement	0.515	0.647	0.013	0.564	0.736	−0.087
Trustworthiness	0.316	0.473	−0.288	0.223	0.426	0.307
Empathy	0.307	0.543	0.106	0.315	0.581	−0.037
Generosity	0.548	0.675	−0.494	0.531	0.712	−0.274
Gratitude	0.231	0.438	0.120	0.452	0.639	−0.109
Emotional forgiveness	0.312	0.553	0.155	0.302	0.548	0.055
Respect	0.443	0.635	0.233	0.424	0.641	0.456
Solidarity	0.611	0.786	−0.156	0.548	0.727	−0.235

Note: The factor loadings have been highlighted to aid in the understanding of the results.

## Data Availability

The data referred to in this article will be made available by the corresponding authors, upon reasonable request.

## Appendix A

A list of moral feelings to be evaluated by experts, along with their respective initial definitions:

1. Authenticity: The feeling of coherence that arises from showing and communicating to others as one honestly feels;
2. Kindness (and goodness): Emotional tendency to ensure that others feel good and are happy;
3. Good humor: Feeling joy in fostering a positive relationship with others;
4. Compassion: The feeling that arises from someone’s suffering and drives one to alleviate their pain, remedy it, or prevent it; to enhance the wellbeing of the one who is suffering;
5. Emotional engagement: Feeling the obligation to fulfill commitments to others to promote an emotional relationship with them;
6. Trustworthiness: The feeling of security conveyed to another person to establish a sincere relationship with them;
7. Empathy: The emotional capacity to see something from another person’s perspective, understand their feelings, and experience emotions towards them;
8. Hope: The feeling of certainty that it is worth the effort to pursue our dreams because we believe they are possible;
9. Fidelity (loyalty): The feeling of coherence evoked when we support the commitments we make to others with our actions;
10. Generosity: The feeling that arises from freely giving another person anything considered necessary for their wellbeing, without expecting anything in return and which is solely for their benefit;
11. Gratitude: The feeling of esteem and acknowledgment of a benefit received or to be received, desiring to reciprocate, and recognizing the importance of others in terms of our emotional wellbeing;
12. Unconditionality: The feeling of accepting another person regardless of who they are or how they behave towards us;
13. Forgiveness: The feeling of relinquishing the desire for retaliation for harm received, not seeking compensation or punishment for someone who has caused harm, while acknowledging their responsibility for it;
14. Respect: The feeling of consideration towards another person, valuing and taking into account their feelings to establish a relationship with them;
15. Solidarity: Feeling the need to collaborate with others by offering help and support in order to seek the common good for the community.

## Appendix B

A list of selected feelings and their definition based on the judges’ indications:

1. Authenticity: The feeling of coherence experienced when showing and communicating to others as one honestly feels (M = 6.18);
2. Kindness (and goodness): The emotional tendency to altruistically ensure that others feel good (M = 6.37);
3. Compassion: The feeling that arises from someone’s suffering and drives one to alleviate their pain, remedy it, or prevent it; to increase the wellbeing of the one who is suffering (M = 6.68);
4. Emotional engagement: Feeling the need to establish an emotional relationship with another person to provide support and assistance (M = 6.56);
5. Trustworthiness: The feeling of security conveyed to another person to establish a sincere relationship with them (M = 6.5);
6. Empathy: The emotional capacity to see something from another person’s perspective, understand their feelings, and experience emotions towards them, while maintaining the emotional distance necessary to help them (M = 6.87);
7. Generosity: The feeling that arises from freely giving another person anything considered necessary for their wellbeing, without expecting anything in return and which is solely for their benefit (M = 6.5);
8. Gratitude: The feeling of esteem and acknowledgment of a benefit received or to be received, desiring to reciprocate, and recognizing the importance of others in regard to our emotional wellbeing (M = 6.5);
9. Emotional forgiveness: The emotional experience associated with the decrease or disappearance of feelings, such as resentment, anger, shame, humiliation, hatred, pain, or bitterness, experienced as a result of feeling harmed or violated by another person (M = 6);
10. Respect: The feeling of consideration towards another person, valuing and taking into account their opinions, actions, and feelings, in order to establish a relationship with them (M = 6.75);
11. Solidarity: Feeling the need to collaborate with others, offering help and support to seek to improve the wellbeing of others (M = 6.62).
